# Investigating the response of paediatric leukaemia‐propagating cells to BCL‐2 inhibitors

**DOI:** 10.1111/bjh.16773

**Published:** 2020-05-25

**Authors:** Paraskevi Diamanti, Benjamin C. Ede, Phoebe EI Dace, William J. Barendt, Charlotte V. Cox, Jeremy P. Hancock, John P. Moppett, Allison Blair

**Affiliations:** ^1^ Bristol Institute for Transfusion Sciences NHSBT Filton Bristol UK; ^2^ School of Cellular and Molecular Medicine University of Bristol Bristol UK; ^3^ Bristol Genetics Laboratory Severn Pathology, North Bristol Trust Bristol UK; ^4^ Bristol Royal Hospital for Children Bristol UK

**Keywords:** ALL, BCL‐2, paediatric ALL, LPC, Navitoclax, Venetoclax, NSG

## Abstract

Relapse of paediatric acute lymphoblastic leukaemia (ALL) may occur due to persistence of resistant cells with leukaemia‐propagating ability (LPC). In leukaemia, the balance of B‐cell lymphoma‐2 (BCL‐2) family proteins is disrupted, promoting survival of malignant cells and possibly LPC. A direct comparison of BCL‐2 inhibitors, navitoclax and venetoclax, was undertaken on LPC subpopulations from B‐cell precursor (BCP) and T‐cell ALL (T‐ALL) cases *in vitro* and *in vivo*. Responses were compared to BCL‐2 levels detected by microarray analyses and Western blotting. *In vitro*, both drugs were effective against most BCP‐ALL LPC, except CD34^−^/CD19^−^ cells. In contrast, only navitoclax was effective in T‐ALL and CD34^−^/CD7^−^ LPC were resistant to both drugs. *In vivo*, navitoclax was more effective than venetoclax, significantly improving survival of mice engrafted with BCP‐ and T‐ALL samples. Venetoclax was not particularly effective against T‐ALL cases *in vivo*. The proportions of CD34^+^/CD19^−^, CD34^−^/CD19^−^ BCP‐ALL cells and CD34^−^/CD7^−^ T‐ALL cells increased significantly following *in vivo* treatment. Expression of pro‐apoptotic BCL‐2 genes was lower in these subpopulations, which may explain the lack of sensitivity. These data demonstrate that some LPC were resistant to BCL‐2 inhibitors and sustained remission will require their use in combination with other therapeutics.

## Introduction

Treatment of childhood acute lymphoblastic leukaemia (ALL) has been very successful in recent years.^1^ However, relapse rates remain high in some subgroups.^2^, ^3^, ^4^ This may be due to the persistence of resistant clones or the emergence of clones following therapy that were not detected at diagnosis.^5^, ^6^ We and others have previously identified ALL cells that have the ability to initiate and maintain the disease in murine models, known as leukaemia‐propagating cells (LPC).^7^, ^8^, ^9^, ^10^, ^11^, ^12^, ^13^, ^14^, ^15^ A common finding, regardless of subtype, was that expression of antigens commonly associated with B‐cell precursor (BCP)‐ALL (CD34, CD10, CD19) or T‐ALL (CD4, CD7) was not a prerequisite for disease‐propagating ability *in vivo*. Indeed, we demonstrated that the frequency of LPC was often higher in CD34^+^/CD19^−^ and CD34^−^ subpopulations than in CD34^+^/CD19^+^ cells in BCP‐ALL, with similar findings using CD34 and CD7 in T‐ALL cases.^12^ Moreover, we have shown that some of these LPC populations, such as CD34^+^/CD19^−^, CD34^+^/CD7^−^ and CD34^−^ cells, can be resistant to treatment *in vivo* and *in vitro*.^8^, ^12^, ^16^ Hence, it is important to assess therapeutic agents on all LPC subpopulations that have capacity to cause relapse.

Bi‐specific T‐cell engagers and chimaeric antigen receptor T cells against CD19 have demonstrated the efficacy of immunotherapeutic approaches to treat ALL.^17^, ^18^, ^19^, ^20^ However, such therapies cannot target LPC that lack CD19 and there is evidence of tumour escape, through lack of targeting, lineage switching and masking of the CD19 epitope.^21^, ^22^ An alternative target of focus is B‐cell lymphoma (BCL)‐2, which has been shown to be overexpressed in >66% of BCP‐ALL cases compared to normal bone marrow (NBM) donors^23^ and is associated with oncogenesis in several cancers.^24^, ^25^ Consequently, BCL‐2 inhibitors have been investigated in ALL.^26^, ^27^, ^28^ ABT‐263 (navitoclax) binds to and inhibits BCL‐2, BCL‐extra large (BCL‐xL) and Bcl‐2‐like protein 2 (BCL‐w) and kills cells in a BCL‐2‐associated X protein (BAX)‐ and BCL‐2‐antagonist/killer protein (BAK)‐dependent manner.^29^, ^30^ However, thrombocytopenia caused by BCL‐xL inhibition, compromises its use.^31^ ABT‐199 (venetoclax) specifically inhibits BCL‐2 allowing release of pro‐apoptotic molecules. It does not bind to BCL‐xL and consequently does not affect platelets.^32^, ^33^

Both navitoclax and venetoclax have been investigated in xenograft models of paediatric ALL with reports of extended survival of NOD.Cg‐*Prkdc^scid^/IL2rγ^tm1Wjl^*/SzJ (NSG) mice using navitoclax.^34^, ^35^ Responses to venetoclax in BCP‐ALL xenografts were more modest,^36^ although results improved when used in combination with vincristine and dexamethasone.^37^ To date, there is no information on the effects of BCL‐2 inhibitors in LPC subpopulations, so the effects on potentially drug‐resistant cells are unknown. Consequently, we have undertaken a direct comparison of navitoclax and venetoclax on unsorted cells and LPC from primary BCP‐ and T‐ALL cases.

## Materials and methods

### Samples

BM cells from children (median age, 7 years; range, 2–17) with BCP‐ (*n* = 20) and T‐ALL (*n* = 11) at diagnosis or relapse were collected with approval of University Hospitals Bristol NHS Trust (Table I). NBM and cord blood (CB) were obtained from healthy donors. Mononuclear cells were cryopreserved as described.^15^, ^16^ Viability on thawing was 69·6 ± 16·1% (ALL) and 73·0 ± 9·3% (normal) samples.

**Table I:** Patient characteristics.

Pt	Sub‐type	Karyotype	Age at diagnosis (years)	Gender	Disease status at biopsy	MRD Risk†	Treatment protocol
1	c‐ALL	Hyperdiploidy	7	M	D	High	UKALL 2003
2	Pre B	t(12;21)	2	F	D	Low	UKALL 2003
3	Pre B	−1	2	F	D	Low	UKALL 2003
4	Pre B	t(9;22)	15	M	D	Low	UKALL 2011
5	Pre B	Hyperdiploidy	2	M	D	Low	UKALL 2003
6	Pre B	46XY	9	M	D	High	UKALL 2011
7	Pre B	t(12;21)	2	F	D	Low	UKALL 2011
8	c‐ALL	t(12;21)	4	M	D	ND	UKALL 2011
9	Pre B	−9, +10, −18	2	F	D	High	UKALL 2003
10	Pre B	46XY	14	F	D	Int	UKALL 2011
11	Pre B	+7, −9, −Y	3	M	D	Low	UKALL 2011
12	c‐ALL	t(12;21)	7	F	D	Low	UKALL 2011
13	c‐ALL	+5	5	F	D	Int	UKALL 2011
14	Pro B	11q23 rearr	15	F	D	High	UKALL 2011
15	Pro B	11q23 rearr	17	F	D	Int	UKALL 2011
16	Pre B	t(12;21)	5	M	D	Low	UKALL 2011
17	Pre B	t(12;21)	3	M	D	Low	UKALL 2011
18	Pre B	PDGFRB rearr, −12p	8	F	D	High	UKALL 2011
19	Pre B	t(1;19)	12	M	D	Low	UKALL 2011
20	c‐ALL	+5	17	M	D	Low	UKALL 2011
21	T‐ALL	+4, +9	15	M	D	High	UKALL 2003
22	T‐ALL	t(11;14)	2	M	R	Low	UKALL 2003
23	T‐ALL	46XY	17	M	D	High	UKALL 2003
24	T‐ALL	−3, −6, −11, +1	2	F	D	ND	UKALL 2011
25	T‐ALL	t(1;14)	3	M	D	Int	UKALL 2011
26	T‐ALL	46XY	6	M	D	Low	UKALL 2011
27	T‐ALL	−4, −9	9	M	D	Low	UKALL 2011
28	T‐ALL	46XY	7	M	D	Int	UKALL 2011
29	T‐ALL	21q22 rearr	3	M	D	ND	UKALL 2011
30	T‐ALL	+7, +9, +14, +mar	4	M	D	Low	UKALL 2011
31	T‐ALL	+ABL1	10	M	D	Int	UKALL 2011

D, diagnosis; Int, intermediate; ND, not determined; R, relapse. ALL, acute lymphoblastic leukaemia; c ‐ALL, common ALL; pre B, B‐cell ALL; pro B, B‐cell ALL; T‐ALL, T‐cell ALL. UKALL 2003; UKALL 2011.

† MRD risk status at day 29 for UKALL 2003 and at week 14 for UKALL 2011.

### Cell sorting

BCP‐ALL cells were stained with antibodies against CD34 (clone 8G12) and CD19 (clone 4G7). T‐ALL cells were stained with anti‐CD34 and anti‐CD7 (clone M‐T701). NBM and CB samples were stained with anti‐CD34 and anti‐CD38 (clone HB7, all BD Biosciences, Oxford, UK). Cells were stained with propidium iodide (PI; Miltenyi Biotec, Bisley, UK) to exclude dead cells and live cells were sorted using a Becton Dickinson Influx cell sorter with BD sortware 1.2.0 (BD Biosciences, see Figure S1).

### In vitro drug sensitivity

ALL and CB cells were treated with increasing concentrations of navitoclax or venetoclax for up to 48 h (Stratech Scientific Limited, Newmarket, UK), according to the manufacturer’s recommendations.^38^, ^39^, ^40^ Apoptosis and viability were assessed by flow cytometry using annexin V–fluorescein isothiocyanate and PI. Half‐maximal inhibitory concentration (IC_50_) was calculated using non‐linear regression analyses of dose response.

### BCL‐2 quantification

BCP‐, T‐ALL and CB cells were stained with anti‐CD34 and one of the following; anti‐CD19 (BCP‐ALL), anti‐CD7 (T‐ALL) or anti‐CD38 (CB) and viability dye eFluor 780 (Biolegend, London, UK), fixed, permeabilised and stained with anti‐human BCL‐2 phycoerythrin (BD Biosciences), according to the manufacturer’s instructions. Samples were analysed using a MACSQuant 10 flow cytometer (Miltenyi Biotec) with FlowJo software Version 7.10 (detailed in the supplemental file).

### Western blotting

BCL‐2 proteins were detected using mouse anti‐human BCL‐2, rabbit anti‐human MCL1 (both New England Biolabs, Hitchin, UK) and mouse anti‐human BCL‐xL (Abcam, Cambridge, UK), all at 1:1000. IgG goat anti‐rabbit‐peroxidase (1:5000, Thermo Fisher Scientific, Loughborough, UK) or IgG sheep anti‐mouse‐peroxidase (1:10 000, GE Healthcare, Little Chalfont, UK) were used as secondary antibodies and levels were detected using luminescence substrate (BioRad, Watford, UK).

### Microarray analyses

Gene expression analysis of BCP‐, T‐ALL and NBM samples was performed using Agilent Whole Human Genome Oligo Microarrays (Waldbronn, Germany), as previously described and detailed in supplementary files.^15^ Microarray data are available in the ArrayExpress database (www.ebi.ac.uk/arrayexpress), accession number E‐MTAB‐4006.

### In vivo studies

ALL was established in NSG mice using primary samples as described previously, and detailed in Data S1.^12^, ^15^ Briefly, once the level of human cells in murine peripheral blood (PB) was ≥0·5%, animals were given BCL‐2 inhibitors (100 mg/kg/day) for 21 days by oral gavage. Separate cohorts of mice were treated with standard chemotherapy or placebo. PB aspirates were monitored weekly by flow cytometry and animals maintained until they began to exhibit symptoms of disease.

### Statistical analyses

Full details are provided in Data S1.

## Results

### Effect of BCL‐2 inhibitors in vitro

**Fig 1:**
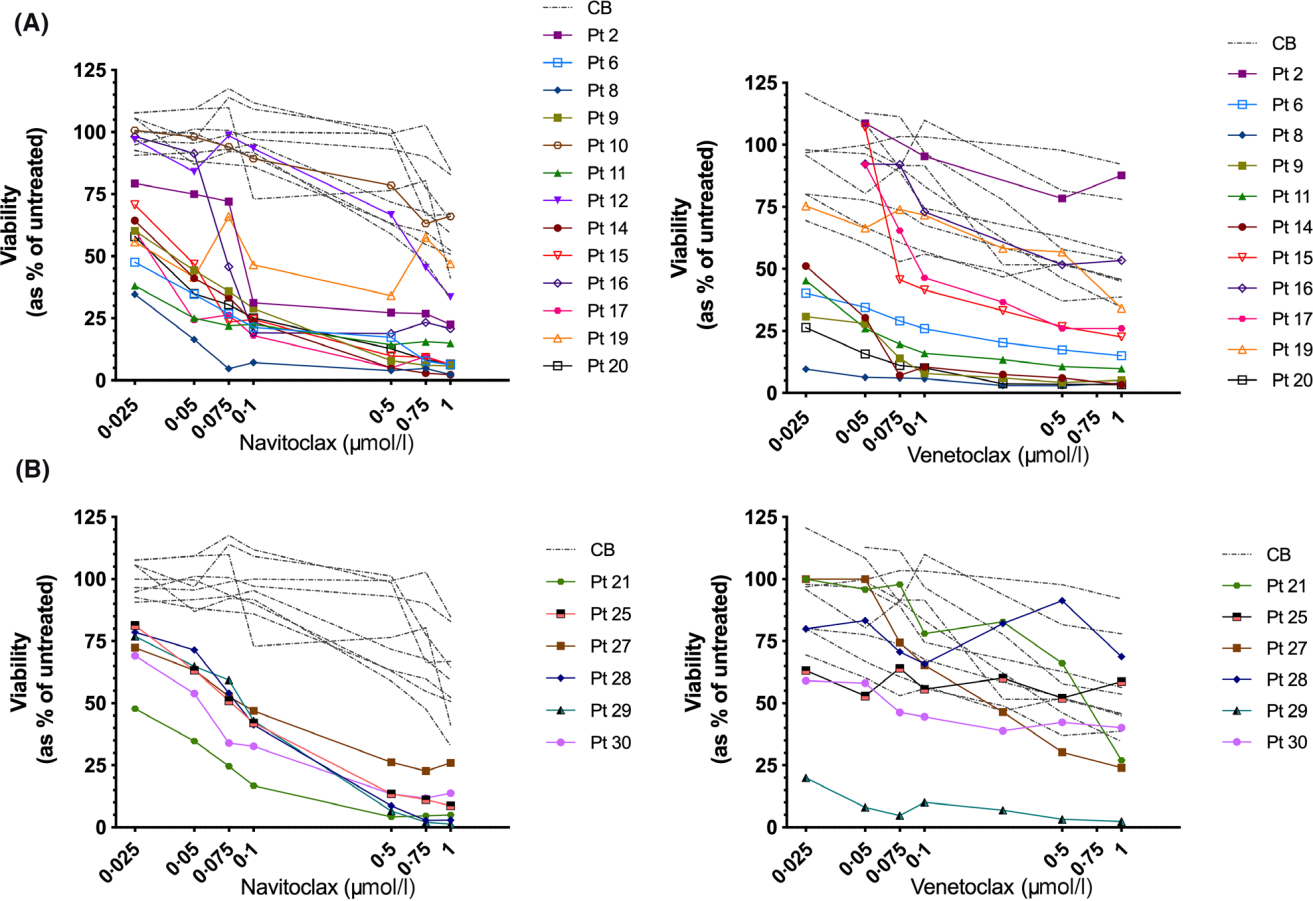
Response of normal and acute lympohoblastic leukaemia (ALL) cells to B‐cell lymphoma‐2 (BCL‐2) inhibitors. (A) Response of B‐cell precursor (BCP) ALL (pts. 2, 6, 8, 9–12, 14–17, 19, 20) and normal CB (*n* = 9) to increasing doses of navitoclax and venetoclax for up to 48 h. (B) Survival of T‐cell (T‐)ALL (pts. 21, 25, 27–30) treated as above and compared to cord blood (CB). Symbols and lines represent individual patient responses. [Colour figure can be viewed at wileyonlinelibrary.com]

A range of responses to the pan‐BCL‐2 inhibitor, navitoclax, and the BCL‐2‐specific inhibitor, venetoclax, were observed (Fig. 1A). Eleven BCP‐ALL cases (pts. 2, 6, 8, 9, 11, 14–17, 19, 20) were sensitive to navitoclax with a mean IC_50_ of 41 nmol/l (*P* ≤ 0·0001 cf. CB) and two were more resistant (pts. 10,12, IC_50_ ≥ 693 nmol/l). Similar results were obtained in a cohort using venetoclax, where eight sensitive cases (pts. 6, 8, 9, 11, 14, 15, 17, 20) had a mean IC_50_ of 35 nmol/l (*P* ≤ 0·0001 cf. CB). Pts. 2, 16 and 19 were more resistant to venetoclax, although all three cases responded to navitoclax. Individual IC_50_ values are shown in Table SI. There was no significant difference in responses between measurable residual disease (MRD) low, intermediate or high risk groups (*P* = 0·52 for navitoclax and *P* = 0·37 for venetoclax).

All six T‐ALL cases responded to navitoclax (mean IC_50_ 69 nmol/l, *P* < 0·0001 cf. CB, Fig 1B). In contrast, five of these cases were resistant to venetoclax (IC_50_ 496 nmol/l). The remaining case (pt. 29) responded to venetoclax at all doses tested (*P* = 0·004). Responses to navitoclax did not differ between MRD groups (*P* = 0·21); however intermediate and high risk samples were significantly more resistant to venetoclax (IC_50_ 908 nmol/l, *P* = 0·02) than low risk cases (IC_50_ 131 nmol/l).

### Comparison of in vitro sensitivity and BCL‐2 protein expression

**Fig 2:**
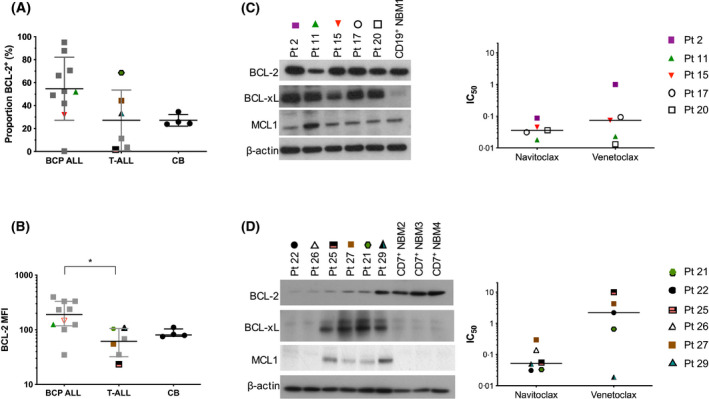
Expression of B‐cell lymphoma‐2 (BCL‐2) in leukaemia and normal haemopoietic cells. (A) Proportion of BCL‐2^+^ cells and (B) median fluorescence intensity of BCL‐2 in 10 B‐cell precursor (BCP) acute lympohoblastic leukaemia (ALL) (pts. 6, 8, 9, 11–15, 18, 19), six T‐cell (T‐)ALL (pts. 21, 25, 27–30) and four cord blood (CB) samples. Symbols represent individual patient samples. Coloured symbols depict samples shown in panels (C) and (D). Lines in (A) represent mean ± standard deviation (SD). Lines in (B) represent median with interquartile range. *, *P* ≤ 0·05. (C, D) Expression of prosurvival proteins in BCP‐ (pts. 2, 11, 15, 17, 20) and T‐ALL (pts. 21, 22, 25–27, 29), respectively. BCP‐ALL samples were compared to CD19^+^ normal bone marrow (NBM) cells, while T‐ALL samples were compared to CD7^+^ NBM cells. The IC_50_ values for each patient sample for navitoclax and venetoclax are depicted in the graphs. Lines represent median values, symbols represent results from individual patient samples.

To determine whether these responses were associated with BCL‐2 expression, flow cytometric analyses were performed. The proportion of BCL‐2^+^ cells was higher in BCP‐ALL cases (60·0 ± 23·0%) compared to T‐ALL (32·8 ± 31·2%, *P* = 0·1) and normal cells (27·2 ± 5·0%, *P* = 0·08, Fig 2A). Likewise, BCL‐2 median fluorescence intensity (MFI) was significantly higher in BCP‐ALL (median 190, range 35–397) than in T‐ALL (62, range 23–109, *P* = 0·02) and >2‐fold higher than normal cells (81, range 75–111, *P* = 0·09, Fig 2B). Western blotting confirmed BCL‐2 expression was higher in BCP‐ than T‐ALL cases and at least equivalent to that in normal CD19^+^ B cells (Fig 2C,D). Of the anti‐apoptotic proteins, BCL‐xL was the most prominent. Myeloid cell leukaemia 1 protein (MCL1 was present in all cases but did not differ from NBM cells, except in pt. 11, where higher levels were observed. BCL‐w could not be detected in this cohort. Interestingly, pt. 2, who had the highest BCL‐2, had the lowest sensitivity to both drugs. In T‐ALL cases, BCL‐2 levels were lower than normal T cells in 5/6 cases and comparable in pt. 29 (Fig 2D). The prosurvival proteins BCL‐xL and MCL1 were expressed in most T‐ALL cases but not in normal CD7^+^ cells. Amongst the samples that were more sensitive to navitoclax, pts. 21, 25 & 29 had elevated levels of BCL‐xL and MCL1. Pt. 29, who had the highest BCL‐2 expression, was the only responder to venetoclax.

### Response of LPC to BCL‐2 inhibitors

**Fig 3:**
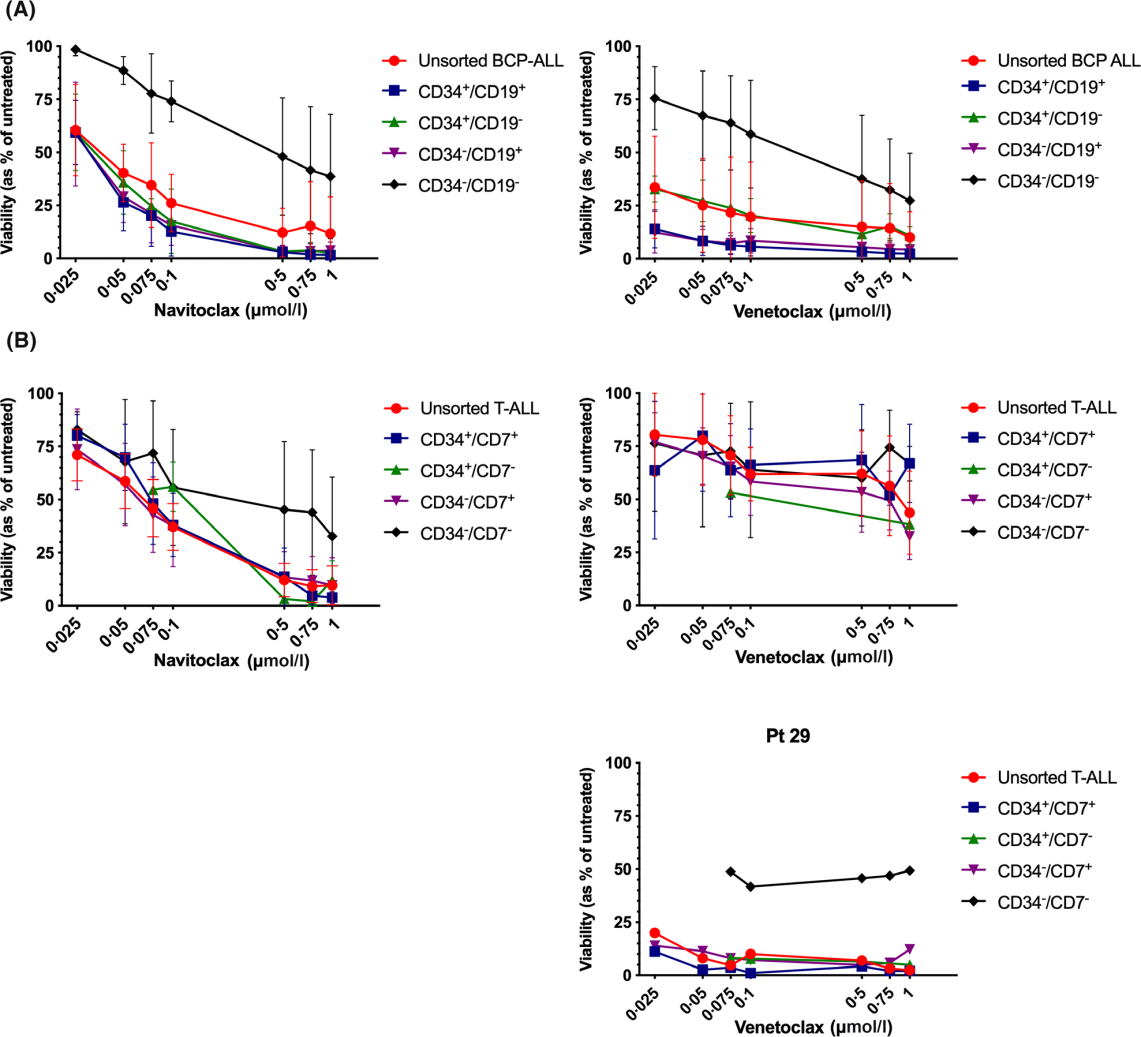
Viability of leukaemia‐propagating cells following B‐cell lymphoma‐2 (BCL‐2) inhibitor treatment. (A) B‐cell precursor (BCP) acute lympohoblastic leukaemia (ALL) cells from six cases (pts. 9, 11, 14, 15, 19, 20) were sorted into four subpopulations using antibodies against CD34 and CD19. Each subpopulation was treated with navitoclax or venetoclax for up to 48 h and cell survival was measured by flow cytometry using annexin V and propidium iodide (PI). (B) Cells from six T‐cell (T‐)ALL cases (21, 25, 27–30) were sorted into four subpopulations using antibodies against CD34 and CD7 and treated with BCL‐2 inhibitors, as above. Unsorted cells from five T‐ALL cases were resistant to venetoclax (pts. 21, 25, 27, 28, 30) and 1one was sensitive (pt. 29, depicted separately). Viability is shown as mean ± SD across samples, expressed as a percentage of untreated controls. [Colour figure can be viewed at wileyonlinelibrary.com]

Both inhibitors were subsequently assessed on recognised LPC subpopulations^7^, ^10^, ^11^, ^12^, ^16^ from a cohort of six BCP‐ALL samples, including responders and less sensitive cases, and from all six T‐ALL cases used previously. Unsorted cells and three of four BCP‐ALL subpopulations (CD34^+^/CD19^+^, CD34^+^/CD19^−^ and CD34^−^/CD19^+^) responded to both inhibitors, with viability reduced to 1·6 ± 1·6% using 1 µmol/l navitoclax (IC_50_ range 18–54 nmol/l) and 2·4 ± 1·6% with venetoclax (IC_50_ range 3–120 nmol/l). In contrast, CD34^−^/CD19^−^ LPC were more resistant to both drugs with 38·7 ± 29·3% cells surviving treatment with navitoclax and 27·4 ± 22·3% with venetoclax at the highest doses (Fig 3A). Individual results are shown in Table SII.

In T‐ALL samples (Fig 3B), unsorted cells and three subpopulations (CD34^+^/CD7^+^, CD34^+^/CD7^−^ and CD34^−^/CD7^+^) responded to navitoclax (IC_50_ range 21–388 nmol/l) but not to the same extent as BCP‐ALL cases. CD34^+^/CD7^+^ cells were the most responsive (3·9 ± 2·7% viable using 1 µmol/l), while CD34^−^/CD7^−^ cells were the least sensitive with 32·7 ± 27·9% surviving the highest dose. As was observed with the unsorted cells (Fig 1B), LPC from five cases were resistant to venetoclax. Only pt. 29 responded, although CD34^−^/CD7^−^ LPC remained the least sensitive.

### Differential expression of BCL‐2 molecules

As navitoclax and venetoclax both affect other BCL‐2 family members, gene expression levels of all anti‐apoptotic and pro‐apoptotic molecules were determined in a subset of cases and data compared to haemopoietic stem cells (HSC). There was no clear trend in upregulated or downregulated genes in ALL samples (Table II, Figures S2–S4). Some LPC had higher levels of anti‐apoptotic genes (BCL‐2 alpha, BCL‐xL and BFL1/A1) than HSC. However, higher levels of pro‐apoptotic genes including BMF (*P ≤ *0·01), BAD and BIM (*P ≤ *0·05), BAX (*P* = 0·05) and BCL2L13 were observed (*P ≤ *0·004). In contrast, BIK (*P* = 0·01) and Caspase (CASP)‐9 (*P* = 0·001) were downregulated. Other genes with significantly different expression in LPC subpopulations are shown in (Table SIII).

**Table II:** Median fold change in expression of BCL‐2 genes in ALL compared to normal BM.

Gene	BCP‐ALL				
	Unsorted	CD34^+^/CD19^+^	CD34^+^/CD19^−^	CD34^−^/CD19^+^	CD34^−^/CD19^−^
Anti‐apoptotic					
BCL‐2 alpha	2·3	1·6	−1·8	1·2	1·6
BCL‐2 beta	−1·2	−2·5	−1·5	−2·1	−1·9
BCL‐xL	−1·7	1·2	1·0	−6·0	−2·0
BCL‐w	3·7	1·8	−1·1	2·0	−2·5
MCL1	2·5	−1·7	−1·2	−1·2	1·4
BFL1/A1	−1·7	2·3	1·5	5·7	2·2
Pro‐apoptotic					
BAX beta	2·2	1·5	1·4	1·3	1·4
BAX sigma	−1·0	1·2	1·3	1·0	1·2
BAK1	1·0	−2·5	−1·3	−1·2	−2·3
BOK	−1·8	−1·7	1·1	−1·6	−1·5
BAD	2·0	−1·6	2·1	−1·7	−2·0
BIK	4·2	−1·8	−4·1	−7·3	−2·6
NOXA	1·4	1·4	1·6	1·2	1·4
HRK	1·3	1·2	−2·0	−1·5	−4·0
PUMA	−1·8	1·1	2·1	−1·1	1·2
BMF	2·8	5·0****	5·5***	3·4**	2·1
BID	3·6	1·5	−1·4	2·4	2·0
BIM var1	−1·0	−1·7	−2·4	−2·6	−1·4
BIM var9	4·7**	2·0	2·5	1·5	1·6
BCL2L13	2·0	1·2	1·2	1·3	1·4
CASP9	−1·6	−3·5	−2·7	−1·9	−3·2

Gene	T‐ALL				
	Unsorted	CD34^+^/CD7^+^	CD34^+^/CD7^−^	CD34^−^/CD7^+^	CD34^−^/CD7^−^
Anti‐apoptotic					
BCL‐2 alpha	2·1	3·5	−1·9	4·5	4·2
BCL‐2 beta	−1·2	−1·3	−2·5	−2·1	−2·4
BCL‐xL	1·4	2·3	1·9	2·7	−1·3
BCL‐w	1·5	1·2	−2·8	2·7	−1·6
MCL1	4·0	−1·5	1·2	−1·2	−1·9
BFL1/A1	1·4	1·8	2·4	2·0	1·5
Pro‐apoptotic					
BAX beta	1·8	−1·0	1·0	1·2	−1·0
BAX sigma	−1·3	1·7*	1·0	1·4	1·6
BAK1	1·1	−2·6	−1·9	−1·7	−3·0
BOK	−1·1	−1·0	−1·3	−1·2	−1·3
BAD	5·8*	1·8	2·3	1·1	−10·0
BIK	3·3	−4·3	−7·3	−13·8	−13·6**
NOXA	−1·1	−1·1	1·1	1·1	−1·2
HRK	1·4	2·3	−1·6	−2·8	−1·5
PUMA	−1·9	1·9	1·1	−1·5	1·8
BMF	−1·9	1·8	−1·1	2·9	1·7
BID	−1·3	1·2	−1·1	−1·1	−10·0
BIM var1	2·7	2·0	−3·2	−2·8	1·2
BIM var9	3·4*	−1·7	−1·3	−1·9	1·3
BCL2L13	1·6	2·1**	1·6	2·3**	−1·5
CASP9	1·3	−4·1	−8·8	−3·6	−3·6***

Unsorted BCP‐ALL (pts 1, 3, 5–7) and T‐ALL (pts 21–25) were compared with healthy BM (*n* = 5). Leukaemia‐propagating subpopulations (CD34^+^/CD19^+^, CD34^+^/CD19^−^, CD34^−^/CD19^+^, CD34^−^/CD19^−^, CD34^+^/CD7^+^, CD34^+^/CD7^−^, CD34^−^/CD7^+^and CD34^−^/CD7^−^) were compared to CD34^+^/CD38^−^ HSC. Data represent median fold change calculated from inverse log_2_ ratios. Data were analysed by ANOVA with Tukey's *post‐hoc* test. *, *P* ≤ 0·05; **, *P* ≤ 0·01; ***, *P* ≤ 0·001; ****, *P* ≤ 0·0001 compared to normal cells. Bold text indicates significant difference from levels in NBM.

ALL, acute lymphoblastic leukaemia; BCL‐2, B‐cell lymphoma‐2; BCL‐w, Bcl‐2‐like protein 2; BCL‐xL, BCL‐extra large; BCP, B‐cell precursor; BM, bone marrow; T‐ALL, T‐cell ALL.

### Navitoclax is superior to venetoclax in vivo

**Fig 4:**
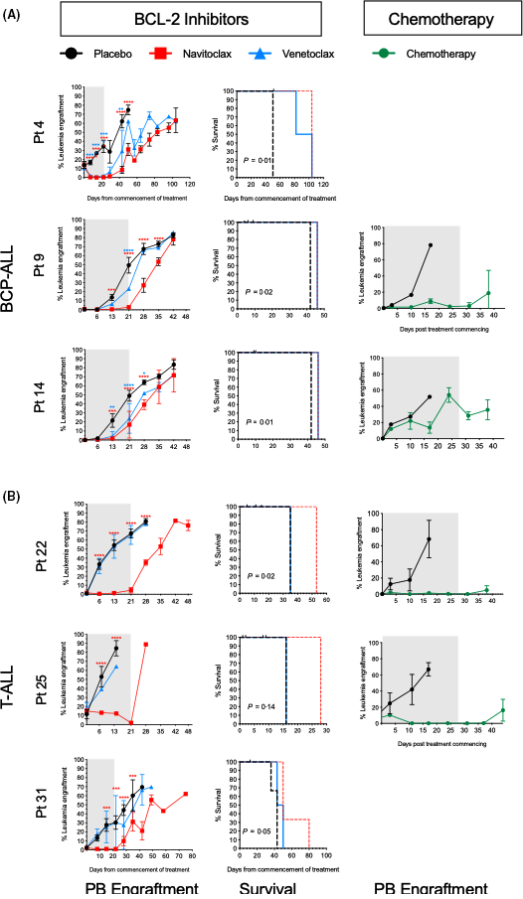
Effect of B‐cell lymphoma‐2 (BCL‐2) inhibitors and standard therapeutics *in vivo*. NSG mice were transplanted with B‐cell precursor (BCP) acute lymphoblastic leukaemia (ALL) (pts. 4, 9, 14) (A) and T‐cell (T‐)ALL (pts. 22, 25, 31) (B). Engraftment levels in peripheral blood (PB) of NSG were monitored weekly by flow cytometry. Treatment with navitoclax or venetoclax or standard induction chemotherapeutics commenced when ≥0·5% human cells were detected in the PB (median time 16 days, range 11–60). At least three animals were used for each treatment group. The left and right panels show the number of leukaemia cells in murine PB over time from commencement of treatment (day 0). Grey boxes represent duration of treatment. Middle panel shows corresponding Kaplan–Meier survival curves for navitoclax‐ and venetoclax‐treated mice. Censored observations are depicted with black tick marks. Curves were compared using the log‐rank test. Data represent mean ± SD. Red asterisks correspond to comparisons between navitoclax‐ and placebo‐treated mice and blue asterisks between venetoclax‐ and placebo‐treated mice. *, *P* ≤ 0·05; **, *P* ≤ 0·01; ***, *P* ≤ 0·001; ****, *P* ≤ 0·0001. [Colour figure can be viewed at wileyonlinelibrary.com]

NSG mice with established leukaemia, from low, intermediate and high risk cases, were treated with navitoclax, venetoclax and, in some cases, standard chemotherapeutic agents (dexamethasone, vincristine, L‐asparaginase ± daunorubicin). In BCP cases, navitoclax treatment decreased leukaemia burden or maintained it at the same level for 7–29 days from treatment start (*P* < 0·0001, Fig 4A). Survival was extended up to 54 days. Using venetoclax, disease burden remained significantly below the levels in placebo groups in pts. 9 & 14 (*P* < 0·0001) during treatment and for a further seven days in pt. 14. However, in pt. 4 (Ph^+^, low risk) disease burden significantly decreased to 2·0 ± 2·0% (*P* < 0·0001) and progression was significantly delayed by three weeks. Survival was improved with both drugs, particularly in pt. 4 (54 days, *P* = 0·01) with more modest improvements in pts. 9 & 14 (four days, *P* ≤ 0·02). These responses were similar to those observed using standard induction therapy for 28 days.

In mice engrafted with T‐ALL samples, navitoclax reduced disease burden and significantly delayed progression in all cases compared to controls (*P ≤ *0·001, Fig 4B). Survival in navitoclax‐treated animals was extended by up to 40 days but disease burden was more effectively reduced using standard chemotherapeutics. Venetoclax had no effect on this T‐ALL cohort.

Analyses of BM from terminated animals revealed an increase in the proportion of CD34^+^/CD19^−^ cells following treatment with navitoclax (8‐fold, *P* ≤ 0·02) and venetoclax (13‐fold, *P* ≤ 0·03) in pt. 4 compared to the proportions in the diagnostic sample (Table III). In pts. 9 & 14, the proportions of CD34^−^/CD19^−^ cells increased significantly following treatment with BCL‐2 inhibitors (*P* ≤ 0·03) and following standard therapy, in pt 14 (*P* = 0·002). In T‐ALL cases, the CD34^−^/CD7^−^ subpopulation, which was just detectable at diagnosis, had increased significantly in pt. 22, following treatment with both inhibitors (*P* ≤ 0·01). Likewise, in pt. 25, the majority of cells recovered from the BM were CD34^−^/CD7^−^, representing a >15‐fold increase (*P* < 0·004) in the proportion of these cells compared to the primary sample inoculated, while the CD34^+^/CD7^+^ and CD34^+^/CD7^−^ cells were significantly depleted (*P* < 0·02). In pt. 31, in contrast, the CD7^−^ subpopulations were most affected by treatment.

**Table III:** Immunophenotype of patient cells at diagnosis and those recovered from treated NSG mice.

Pt. ID	Time point	Proportion of nucleated cells (%)			
		CD34^+^/CD19^+^	CD34^+^/CD19^−^	CD34^−^/CD19^+^	CD34^−^/CD19^−^
BCP‐ALL					
4	Diagnosis	85	0·7	7	7
	Navitoclax	72 ± 11	6 ± 3*	21 ± 12	2 ± 1
	Venetoclax	76 ± 1	10 ± 6*	13 ± 7	1 ± 0·2
9	Diagnosis	1·8	0·2	93·9	4·1
	Navitoclax	1 ± 1	0·1 ± 0·1	92 ± 1	8 ± 0·5*
	Venetoclax	1 ± 1	0·2 ± 0·1	86 ± 1	13 ± 1*
	Std Chem	0·5 ± 0·4*	0·5 ± 0·2	52 ± 41	47 ± 41
14	Diagnosis	25	0·5	72·6	1·9
	Navitoclax	9 ± 2	2 ± 2	78 ± 11	12 ± 9*
	Venetoclax	11 ± 5	1 ± 0·4	77 ± 4	10 ± 4*
	Std Chem	0·9 ± 0·1***	0·1 ± 0·01	87 ± 0·7	12 ± 0·9**
		**CD34^+^/CD7^+^**	**CD34^+^/CD7** ^−^	**CD34** ^−^ **/CD7^+^**	**CD34** ^−^ **/CD7** ^−^
T‐ALL					
22	Diagnosis	0·1	0	99	0·05
	Navitoclax	0	0	93 ± 1	7 ± 1**
	Venetoclax	0	0	85 ± 5	15 ± 5**
	Std Chem	0·9 ± 0·03	0·1 ± 0·1	77 ± 14	22 ± 14
25	Diagnosis	20·1	0·3	75·2	4·4
	Navitoclax	0·2 ± 0·2**	0·4 ± 0·4	31 ± 2*	68 ± 1**
	Venetoclax	0	0	33*	67**
	Std Chem	0·6 ± 0·1***	0·4 ± 0·1	68 ± 9	31 ± 9**
31	Diagnosis	2	14	34	50
	Navitoclax	9 ± 5	2 ± 2	67 ± 27	22 ± 17
	Venetoclax	28 ± 13	2 ± 1	65 ± 22	5 ± 3

Data shows the immunophenotype of primary patient samples at diagnosis and the immunophenotype of cells recovered from the bone marrow of NSG mice engrafted with these patient samples then treated with navitoclax, venetoclax or standard chemotherapeutics (Std Chem: dexamethasone, vincristine, L‐asparaginase ± daunorubicin). Bold text indicates significant difference from inoculated primary sample (*, *P* < 0·05; **, *P* ≤ 0·01; ***, *P* ≤ 0·001).

ALL, acute lymphoblastic leukaemia; BCP, B‐cell precursor; T‐ALL, T‐cell ALL.

## Discussion

Over the last decade there has been considerable interest in BCL‐2 inhibitors for the treatment of cancers. However, their efficacy has not been assessed on LPC subpopulations, which have variable repopulating capacity,^10^, ^11^, ^12^, ^13^ and some are refractory to treatment with conventional (dexamethasone, vincristine) and novel therapeutics, such as parthenolide and Hsp90 inhibitors.^8^, ^12^, ^16^ This is the first report directly comparing the sensitivity of paediatric BCP‐ and T‐ALL samples and their respective LPC subpopulations to navitoclax and venetoclax.

BCL‐2, BCL‐xL and other BCL‐2 family proteins were differentially expressed in ALL pts., resulting in a range of responses to BCL‐2 inhibitors. In BCP‐ALL, 8/11 samples were sensitive to both drugs *in vitro* and three cases responded to navitoclax only. In contrast, all six T‐ALL cases responded to navitoclax but only one case responded to venetoclax.

Most BCP LPC subpopulations responded to both drugs, but more so navitoclax, with the exception of CD34^−^/CD19^−^ cells. T‐ALL LPC were also responsive to navitoclax, although CD34^−^/CD7^−^ LPC were the least sensitive. In contrast, venetoclax was not effective against T‐ALL LPC, with the exception of one pt. where the drug killed most LPC, with limited effects on the CD34^−^/CD7^−^ subpopulation. Our varied findings *in vitro* are in agreement with reports on the activity of venetoclax in ALL cell lines and patient derived xenograft (PDX) samples,^36^, ^41^ acute myeloid leukaemia cell lines,^36^, ^42^ and of navitoclax in ALL cell lines.^34^ The lack of response of T‐ALL cases to venetoclax also concurs with the findings by of Changaile *et al.*, who reported that only the early T‐cell precursor (ETP) subtype was sensitive to venetoclax, indicating this phenotype may be BCL‐2‐dependent.^43^

The reason why a sample responds to navitoclax and not venetoclax may be due to varied expression of BCL‐2 targets. All BCP cases investigated had high levels of BCL‐2, BCL‐xL and MCL1, in accordance with previous reports,^26^, ^35^ and responded to navitoclax. Likewise, T‐ALL cases that responded to navitoclax had higher levels of BCL‐xL and/or MCL1. BCL‐2 expression was higher in BCP‐ than in T‐ALL samples, which may explain why more BCP‐ALL samples responded to venetoclax. Indeed, venetoclax sensitivity in BCP‐ALL has been linked to high expression of BCL‐2 and lower expression of MCL1.^41^ In the present study, venetoclax resistance in T‐ALL may be associated with low levels of BCL‐2.^36^, ^37^ The single T‐ALL case that responded to both drugs was the only one with high BCL‐2, although it was not ETP subtype.

In LPC, the moderately higher levels of BCL‐2 in CD34^−^/CD19^−^ and CD34^−^/CD7^−^ cells seem at odds with the lack of response of these cells. However, inhibition of one BCL‐2 protein can cause partner swapping and change the original dependence of the cell.^44^ Upregulation of pro‐apoptotic genes, BAD and BIM in particular, may also be required to sensitise cells to BCL‐2 inhibitors.^41^, ^42^, ^45^ However, both were downregulated in CD34^−^/CD19^−^ cells. The pro‐apoptotic gene BMF was significantly upregulated in three of four BCP LPC populations, which might explain the higher sensitivity of those LPC compared to CD34^−^/CD19^−^ cells. Overexpression of CTLA4, ITGB2 and MAP2K7, together with lack of expression of CHEK2 may also explain the resistance observed in this subpopulation.^46^, ^47^

In T‐ALL LPC, higher levels of BCL‐2, BCL‐xL, BFL1/A1, PAK2 and tumour suppressor activated protein C (APC) were found. Increased expression of such prosurvival proteins may have reduced the efficacy of venetoclax, as has been reported in other malignancies.^36^, ^45^, ^48^, ^49^ However, high levels of prosurvival proteins did not correlate with venetoclax insensitivity in a study on BCP PDX samples.^41^ It is possible that the very low levels of pro‐apoptotic BAD, BIK and CASP‐9 genes in CD34^−^/CD7^−^ cells may contribute to resistance.

Navitoclax has been reported to have broad efficacy in xenograft models of paediatric ALL, with survival extended by 25–30 days,^34^, ^35^ with more modest effects using venetoclax.^36^ In our direct comparison, the majority of samples responded to navitoclax *in vivo*, with extended survival observed in both subtypes. While venetoclax improved survival in BCP engrafted mice, it was ineffective on those engrafted with T‐ALL. In accordance with reports to date, disease burden increased on withdrawal of treatment.^34^, ^35^, ^36^, ^37^ The lack of response of T‐ALL cases to venetoclax concurs with previous reports,^36^ although Seyfried *et al.* reported delayed progression in one of three PDX samples.^41^ The superior effects of navitoclax *in vivo* support the findings of Khaw *et al.*,^36^ and concur with our findings *in vitro*. However, in two BCP‐ALL cases (intermediate and high risk), promising responses to both drugs *in vitro* were not confirmed *in vivo*, where effects were transient at best.

Since the LPC populations only had moderate responses to navitoclax *in vitro*, with T‐ALL LPC largely unaffected by venetoclax, and sustained remission in treated mice was lacking, direct *in vivo* investigations using these inhibitors on LPC were not pursued. However, the proportions of LPC recovered from murine BM were compared to those in the inoculated patient samples. Interestingly, in intermediate and high risk BCP cases, the proportions of CD34^−^/CD19^−^ cells increased significantly following treatment with BCL‐2 inhibitors and standard therapeutics, while CD34^+^/CD19^−^ cells increased in the low risk case. This concurs with our findings *in vitro* and provides robust functional evidence of resistance in the CD34^−^/CD19^−^ subpopulation. Likewise, a significant increase in CD34^−^/CD7^−^ cells was observed in T‐ALL cases, analogous to the poor *in vitro* response observed in this subpopulation, with a corresponding decrease in the proportion of CD7^+^ cells. We have previously shown that some LPC subpopulations (CD34^+^/CD19^−^ in BCP‐ALL and CD34^+^/CD7^−^ and CD34^−^ in T‐ALL), that have self‐renewal capacity over several generations in NSG mice, are resistant to therapy.^8,12,16^ The results in this study add to the evidence of refractoriness in these subpopulations, which would also be resistant to chimaeric antigen receptor (CAR) T cells directed at CD19 or CD7. Therefore, alternative therapeutic options will be required to eradicate these subpopulations.

Collective results indicate that BH3 mimetics may not be effective as single agents for paediatric leukaemias, especially as the LPC subpopulations expressed BCL‐2 genes at various levels. An alternative approach may be to combine BH‐3 mimetics with inhibitors of survival pathways (e.g. tyrosine kinase inhibitors) to increase apoptosis with potentially fewer side effects than using conventional cytotoxic therapies.

## Funding information

This article presents independent research commissioned by the National Institute for Health Research (NIHR) under its Programme Grants scheme (RP‐PG‐0310‐1003) and its Blood and Transplant Unit (NIHR BTRU) in stem cell transplantation and immunotherapy at University of Bristol in partnership with NHS Blood and Transplant. Support was also provided by the Wellcome Trust Institutional Strategic Support Fund through the Elizabeth Blackwell Institute, University of Bristol and NHS Blood and Trasnplant.

## Author contributions

PD conceived study, designed and performed experiments and wrote the report. BCE processed samples, designed and performed experiments and commented on the report. PEID performed experiments and commented on the report. WJB performed experiments and commented on the report. CVC processed samples and commented on the report. JPH performed MRD testing on patient samples and commented on the report. JPM facilitated sample collection, collated the clinical data information and commented on the report. AB conceived and designed the study, performed *in vivo* experiments and wrote the report.

## Conflict of interests

The authors declare to have no potential conflicts of interest regarding the present work.

## Supporting information


**Data S1.** Supplemental methods.
**Table SI.** IC_50_ values of individual patient samples following BCL‐2 inhibitor treatment.
**Table SII.** Proportions of LPC subpopulation in individual samples and IC_50_ values following BCL‐2 inhibitor treatment.
**Table SIII.** Median fold change in expression of genes in acute lymphoblastic leukaemia (ALL) LPC compared to normal HSC.
**Fig S1.** Gating strategy for sorting leukaemia subpopulations.
**Fig S2.** Expression of anti‐apoptotic and pro‐apoptotic BCL‐2 genes in BCP acute lymphoblastic leukaemia (ALL) and normal cells.
**Fig S3.** Microarray analysis of anti‐apoptotic and pro‐apoptotic BCL‐2 genes in T‐cell acute lymphoblastic leukaemia (T‐ALL) and normal cells.
**Fig S4.** Functional grouping of differentially expressed genes.
