# Supplementary material for: Investigating the response of paediatric leukaemia‐propagating cells to BCL‐2 inhibitors
Source: Br J Haematol. 2020 May 25;192(3):577–88. doi: 10.1111/bjh.16773 (PMC8237230; doi:10.1111/bjh.16773)
Supplement: Supplementary file 1 — Data S1. Supplemental methods. Table SI. IC50 values of individual patient samples following BCL‐2 inhibitor treatment. Table SII. Proportions of LPC subpopulation in individual samples and IC50 values following BCL‐2 inhibitor treatment. Table SIII. Median fold change in expression of genes in acute lymphoblastic leukaemia (ALL) LPC compared to normal HSC. Fig S1. Gating strategy for sorting leukaemia subpopulations. Fig S2. Expression of anti‐apoptotic and pro‐apoptotic BCL‐2 genes in BCP acute lymphoblastic leukaemia (ALL) and normal cells. Fig S3. Microarray analysis of anti‐apoptotic and pro‐apoptotic BCL‐2 genes in T‐cell acute lymphoblastic leukaemia (T‐ALL) and normal cells. Fig S4. Functional grouping of differentially expressed genes. [file BJH-192-577-s001.docx]

**Supplemental Methods**

**Microarray Analyses**

Gene expression analysis of BCP-, T-ALL and NBM samples was performed using Agilent Whole Human Genome Oligo Microarrays. Differential gene expression analysis was performed on comparisons between LPC from BCP ALL and T-ALL versus normal haemopoietic stem cells (HSC) and unsorted BCP ALL and T-ALL versus unsorted NBM. Functional grouping analysis was performed using annotations derived from Gene Ontology ([www.geneontology.org/](http://www.geneontology.org/)). Microarray data are available in the ArrayExpress database ([www.ebi.ac.uk/arrayexpress](https://www.ebi.ac.uk/arrayexpress/)) under accession number E-MTAB-4006.

**BCL-2 quantification**

BCP-, T-ALL and CB cells were stained with anti-CD34 (clone 8G12), anti-CD19 (clone 4G7 for BCP-ALL), anti-CD7 (clone M-T701 for T-ALL), anti-CD38 (clone HB7 for CB) and viability dye eFluor 780 (Biolegend, London, UK), fixed, permeabilised and stained with anti-human BCL-2 Phycoerythrin (BD Biosciences), prior to flow cytometric analysis. Lymphoid populations were selected by gating on low forward and low side scatter events. Dead cells were excluded by gating out efluor780^+^ cells. LPC subsets were analysed further using CD34 and CD19 (BCP-ALL) or CD34 and CD7 (T-ALL) and measuring BCL-2 expression in each of the 4 subsets for BCP- and T-ALL.

**In vivo analyses**

ALL was established in NSG mice using primary samples. Once the level of human cells in murine PB was ≥0.5%, animals were given BCL-2 inhibitors (100mg/kg/day) in 10% ethanol (Eth; Fisher Scientific, Loughborough, UK), 30% polyethylene glycol 400 (PEG400; Sigma-Aldrich) and 60% Phosal 50 PG (Ph50PG; Lipoid AG, Steinhausen, Switzerland) for 21 days by oral gavage. Placebo groups received Eth/PEG400/Ph50PG. Separate cohorts of mice were treated with standard chemotherapy. Mice engrafted with measurable residual disease (MRD) low risk samples were treated with 0.15mg/kg vincristine (Selleckchem, München, Germany) intraperitoneally (ip) 1x/week, dexamethasone (Sigma-Aldrich) 5mg/kg/day ip and L-asparaginase (Apollo Scientific, Stockport, UK) 1000 KU/kg/day ip, 5 days per week. NSG engrafted with intermediate and high risk samples received the above plus daunorubicin (Selleckchem) intravenously 2.5mg/kg 1x/week. Animals were monitored and maintained until they began to exhibit symptoms of disease. BM cells were stained with antibodies against CD7, CD19, CD34, CD45 and murine CD45 (all BD Biosciences) and analysed by flow cytometry.

**Statistical analyses**

One-way analysis of variance (ANOVA), with Tukey’s post-hoc testing was used to compare responses to BCL-2 inhibitors, median fluorescence intensity (MFI) and proportions of BCL-2^+^ cells between ALL cells and normal cells. It was also used to compare expression of BCL-2 family members in different LPC subpopulations. Two-way ANOVA, followed by Tukey post-hoc testing, was used to compare drug responses between ≥3 LPC subpopulations and engraftment levels of untreated and drug-treated cohorts. Kaplan-Meier survival curves were compared using log-rank tests.

**Supplemental Table i IC_50_ values of individual patient samples following BCL-2 inhibitor treatment.**

| **Pt. ID** | IC_50_ (nM) | |
| --- | --- | --- |
| **BCP ALL** | Navitoclax | Venetoclax |
| 2 | 87 | NR |
| 6 | 29 | 22 |
| 8 | 17 | 6 |
| 9 | 45 | 18 |
| 10 | NR | - |
| 11 | 18 | 23 |
| 12 | 693 | - |
| 14 | 43 | 27 |
| 15 | 45 | 74 |
| 16 | 73 | NR |
| 17 | 31 | 93 |
| 19 | 30 | 413 |
| 20 | 36 | 13 |
|  |  |  |
| **T-ALL** | Navitoclax | Venetoclax |
| 21 | 30 | 697 |
| 25 | 78 | NR |
| 27 | 82 | 208 |
| 28 | 83 | 1026 |
| 29 | 86 | 10 |
| 30 | 52 | 54 |

IC_50_ values (nM) derived from non-linear regression analysis of viability data, using the variable slope model. NR: IC_50_ not reached.

**Supplemental Table ii Proportions of LPC subpopulation in individual samples and IC_50_ values following BCL-2 inhibitor treatment**

| **BCP- ALL** | **Unsorted** | **CD34^+^/CD19^+^** | | **CD34^+^/CD19^-^** | | **CD34^-^/CD19^+^** | | **CD34^-^/CD19^-^** | |
| --- | --- | --- | --- | --- | --- | --- | --- | --- | --- |
|  | Navitoclax |  |  |  |  |  |  |  |  |
| Pt. ID | IC_50_^*^ | Proportion of cells | IC_50_ | Proportion of cells | IC_50_ | Proportion of cells | IC_50_ | Proportion of cells | IC_50_ |
| 9 | 45 | 1.8% | - | 0.2% | - | 93.9% | 53 | 4.1% | - |
| 11 | 18 | 28.0% | 39 | 27.9% | 38 | 38.5% | 23 | 5.6% | 105 |
| 14 | 43 | 25.0% | 54 | 0.5% | - | 72.6% | 45 | 1.9% | - |
| 15 | 45 | 37.8% | 21 | 0.7% | - | 58.9% | 18 | 2.6% | 311 |
| 19 | 30 | 17.1% | 28 | 29.1% | 37 | 44.3% | 32 | 9.5% | NR |
| 20 | 36 | 89.8% | 27 | 0.8% | - | 2.4% | 19 | 7.0% | 552 |
|  |  |  |  |  |  |  |  |  |  |
|  | Venetoclax |  | |  | |  | |  | |
| 9 | 18 |  | - |  | - |  | 120 |  | - |
| 11 | 23 |  | 10 |  | 13 |  | 7 |  | 39 |
| 14 | 27 |  | 7 |  | - |  | 5 |  | - |
| 15 | 74 |  | 3 |  | - |  | 1 |  | 102 |
| 19 | 413 |  | 12 |  | 19 |  | - |  | 1234 |
| 20 | 13 |  | 9 |  | - |  | 9 |  | 225 |
|  |  |  | |  | |  | |  | |
| **T-ALL** | **Unsorted** | **CD34^+^/CD7^+^** | | **CD34^+^/CD7^-^** | | **CD34^-^/CD7^+^** | | **CD34^-^/CD7^-^** | |
|  | Navitoclax |  | |  | |  | |  | |
| Pt. ID | IC_50_ | Proportion of cells | IC_50_ | Proportion of cells | IC_50_ | Proportion of cells | IC_50_ | Proportion of cells | IC_50_ |
| 21 | 30 | 0.4% | - | 0.04% | - | 98.9% | 21 | 0.7% | - |
| 25 | 78 | 20.1% | 388 | 0.3% | - | 75.2% | 75 | 4.4% | 928 |
| 27 | 82 | 0.1% | - | 1.5% | - | 94.8% | 151 | 3.6% | - |
| 28 | 83 | 16.6% | 99 | 0.7% | - | 77.1% | 93 | 5.6% | 63 |
| 29 | 86 | 79.2% | 87 | 0.3% | 84 | 19.0% | 45 | 1.5% | 75 |
| 30 | 52 | 6.9% | 45 | 0.5% | - | 89.0% | 68 | 3.6% | NR |
|  |  |  |  |  |  |  |  |  |  |
|  | Venetoclax |  |  |  |  |  |  |  |  |
| 21 | 697 |  | - |  | - |  | 860 |  | - |
| 25 | NR |  | NR |  | - |  | 71 |  | NR |
| 27 | 208 |  | - |  | - |  | 106 |  | - |
| 28 | 1026 |  | NR |  | - |  | 949 |  | 35 |
| 29 | 10 |  | 13 |  | 16 |  | 7 |  | 74 |
| 30 | 54 |  | 5 |  | - |  | 72 |  | NR |

***IC_50_ values (nM) derived from non-linear regression analyses of viability data, using the variable slope model. NR: IC_50_ not reached; -: insufficient data points to calculate IC_50._

**Supplemental Table iii Median fold change in expression of genes in ALL LPC compared to normal HSC**

| **BCP-ALL** |  | | | | |
| --- | --- | --- | --- | --- | --- |
| **Gene** | **Unsorted** | **CD34^+^/CD19^+^** | **CD34^+^/CD19^-^** | **CD34^-^/CD19^+^** | **CD34^-^/CD19^-^** |
| CHEK2 | -2.6 | -2.9 | -3.3 | -2.8 | **-9.2**** |
| CTLA4 | 6.9 | 1.3 | -1.3 | 1.8 | **81.9***** |
| PXN | -2.1 | -1.6 | -2.7 | -3.6 | **3.6*** |
| EPHA4 | 3.7 | 1.4 | 1.5 | 1.8 | **6.2**** |
| TFPT | 1.3 | 1.5 | 1.9 | 1.5 | **2.3****** |
| ITGB2 | 1.4 | 3.0 | 1.4 | 2.7 | **11.8**** |
| MAP2K7 | -1.4 | 1.7 | 1.2 | 2.6 | **5.3**** |
| **T-ALL** |  | | | | |
| **Gene** | **Unsorted** | **CD34^+^/CD7^+^** | **CD34^+^/CD7^-^** | **CD34^-^/CD7^+^** | **CD34^-^/CD7^-^** |
| APC | -1.1 | -1.1 | -2.0 | 3.0 | **5.5**** |
| FANCD2 | -3.4 | 3.3 | 11.9 | 2.1 | **-4.5*** |
| PAK2 | 1.3 | 1.7 | 1.6 | 1.7 | **3.4**** |
| TUBG1 | **4.4*** | -1.9 | 1.3 | -3.3 | **-13.1**** |

CHEK2, check kinase 2; CTLA4, cytotoxic T-lymphocyte associated protein 4; PXN, paxillin; EPHA4, ephrin type-A receptor 4; TFGTP, transcription factor 3 fusion partner; ITGB2, integrin beta chain-2; MAP2K7, mitogen-activated protein kinase kinase 7; APC, adenomatous polyposis coli; FANCD2, Fanconi anemia group D2; PAK2, protein 21 activated kinase 2; TUBG1, tubulin γ1.

**
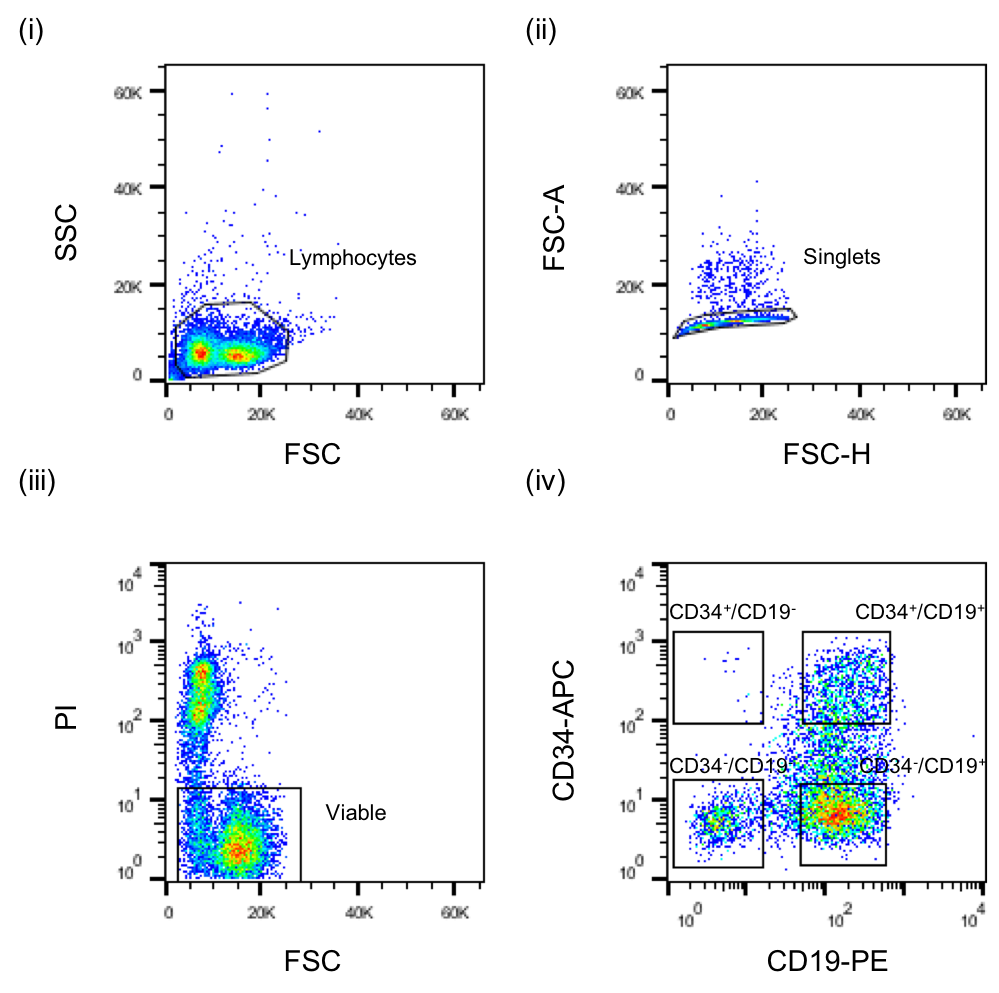
**

**Supplemental Figure 1 Gating strategy for sorting leukemia subpopulations**

Sequential gating strategy to sort primary BCP ALL samples into CD34^+^/CD19^+^, CD34^+^/CD19^-^, CD34^-^/CD19^+^ and CD34^-^/CD19^-^ subpopulations. Cells were stained with CD34-APC, CD19-PE and PI and gated (i) on bases of FSC and SSC scatter, (ii) exclusion of doublets, (iii) exclusion of PI^+^ cells and (iv) on the 4 populations of interest.

**
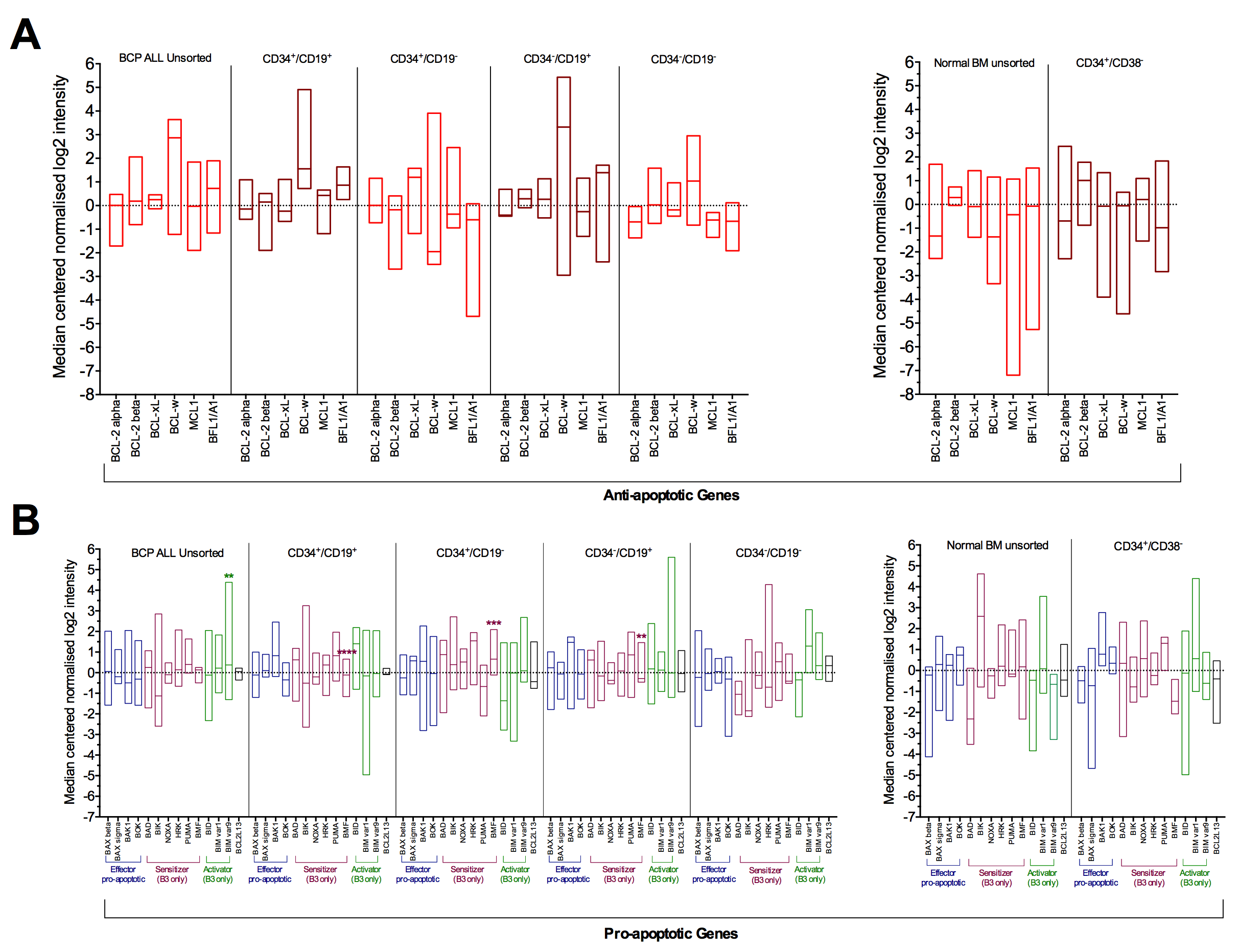
**

**Supplemental Figure 2 Expression of anti-apoptotic and pro-apoptotic BCL-2 genes in BCP ALL and normal cells**

Gene expression of BCL-2 family members in BM samples from 5 BCP ALL (pts 1,3,5-7) and from 5 healthy donors were analyzed using Agilent Whole Genome Oligo microarrays. Data shows side by side comparisons of median centered normalized log2 signal intensities. (A) Anti-apoptotic BCL-2, BCL-xL, MCL1, BCL-w, BFL1/A1 and (B) pro-apoptotic BCL-2 effectors BAX, BAK and BOK (blue bars), sensitizers BAD, BIK, NOXA, HRK, PUMA, BMF (magenta bars) and activators BID and BIM (green bars) are shown. BCP-ALL cases were sorted based on expression or lack of expression of CD34 and CD19 and CD34^+^/CD38^-^ HSC were sorted from NBM. Boxes represent range of expression, horizontal lines depict the median. Results were analyzed by one-way ANOVA with Tukey’s post-hoc testing. *P≤0.05, ** P≤0.01, ***P≤0.001 compared to normal cells.


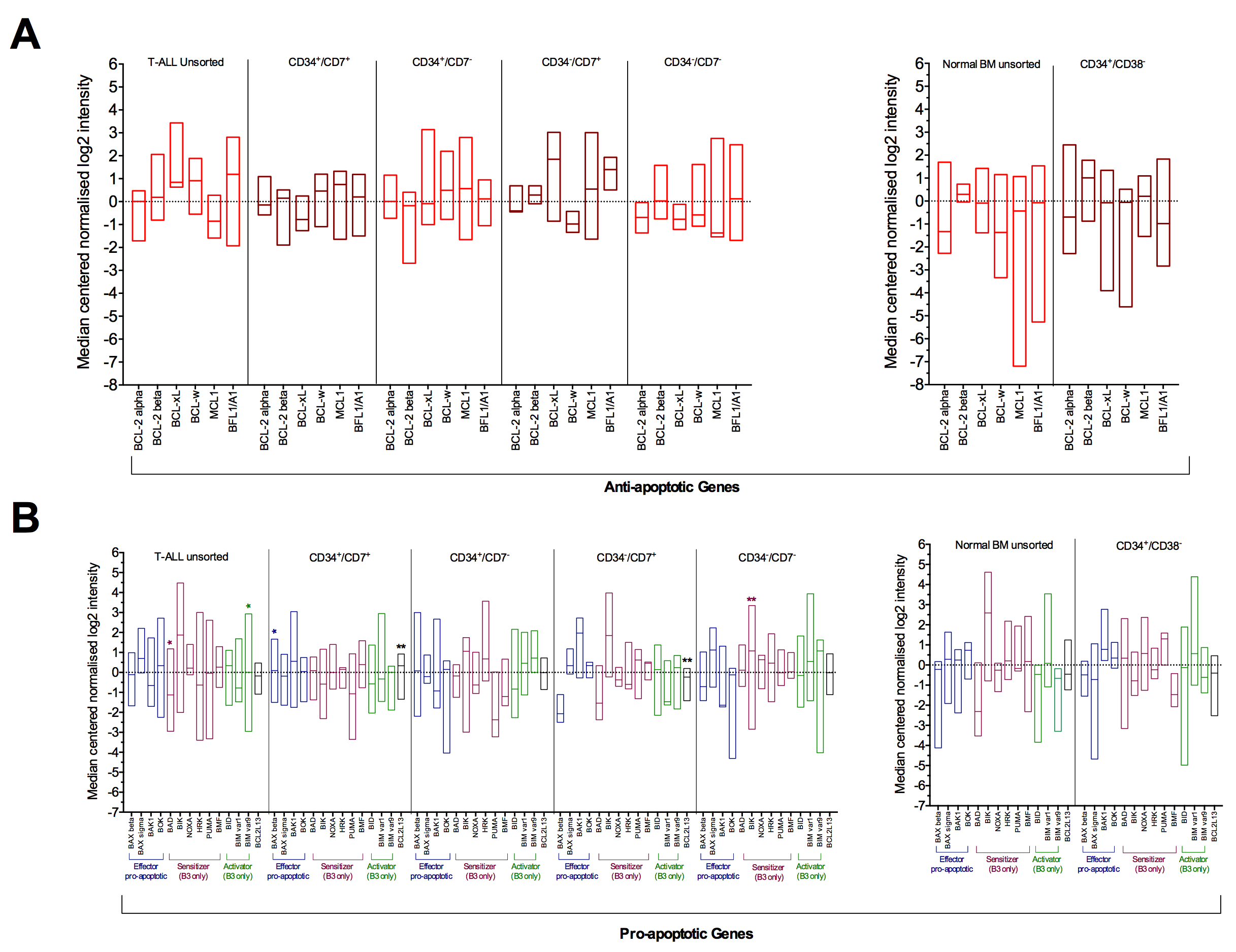


**Supplemental Figure 3 Microarray analysis of anti-apoptotic and pro-apoptotic BCL-2 genes in T-ALL and normal cells**

(A) Expression of anti-apoptotic genes BCL-2, BCL-xL, MCL1, BCL-w, BFL1/A1 and (B) pro-apoptotic BCL-2 effectors BAX, BAK and BOK (blue bars), sensitizers BAD, BIK, NOXA, HRK, PUMA, BMF (magenta bars), activators BID and BIM (green bars) and BCL2L13 in BM samples from 5 T-ALL cases (pts 22-26) and from 5 healthy donors was analyzed using Agilent Whole Genome Oligo microarrays. Data shows side by side comparisons of median centered normalized log2 signal intensities. T-ALL cases were sorted based on expression or lack of expression of CD34 and CD7 and CD34^+^/CD38^-^ HSC were sorted from NBM. Boxes represent the range of expression, horizontal lines depict the median. Results were analyzed by one-way ANOVA with Tukey’s post-hoc testing. *P≤0.05, ** P≤0.01, ***P≤0.001 compared to normal cells.


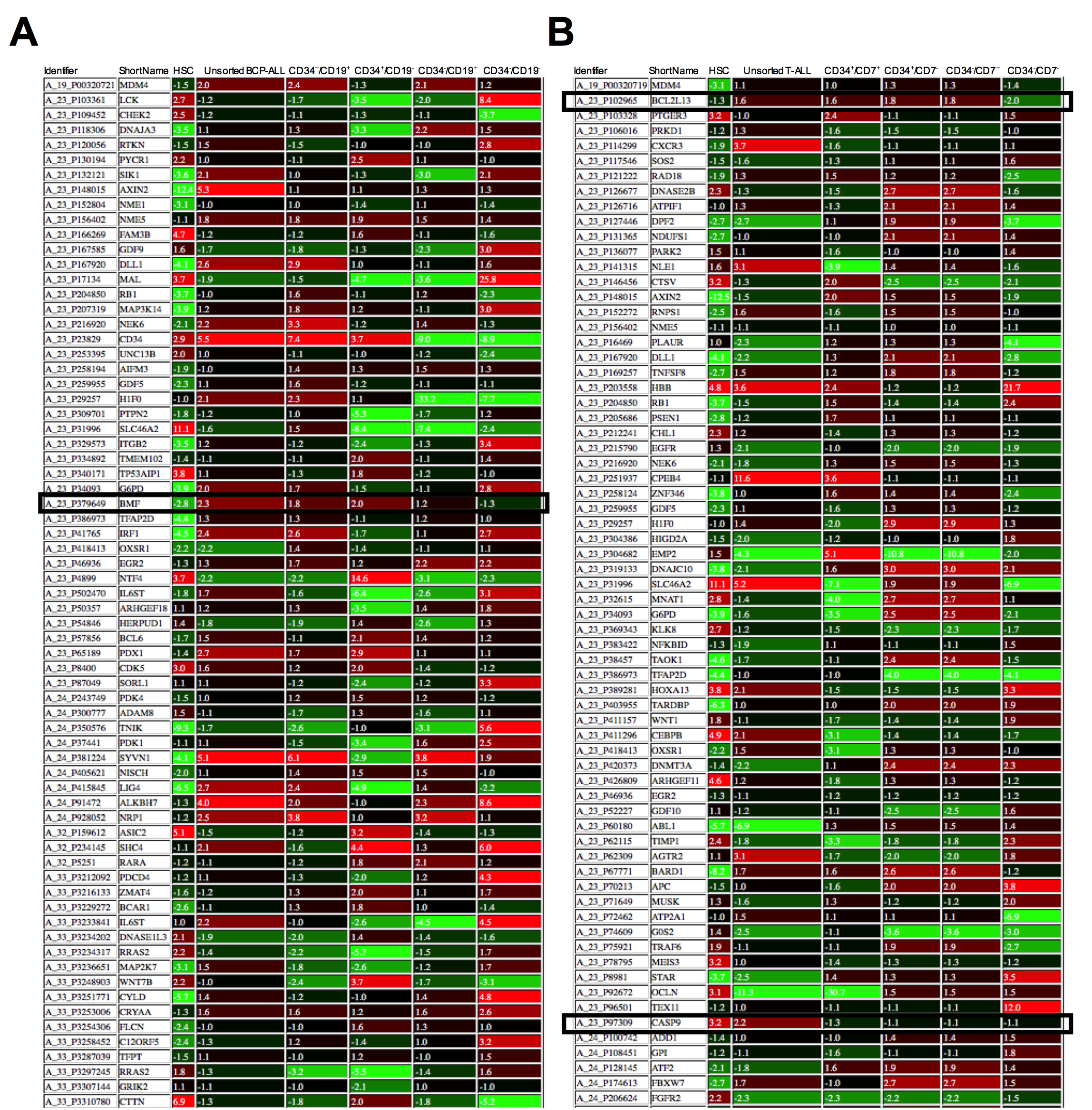


**Supplemental Figure 4 Functional grouping of differentially expressed genes**

Examples of significantly differentially expressed genes involved in cell death in BCP-ALL (A) and T-ALL(B) subpopulations compared to normal HSC. Analyses were based on 5 BCP ALL, 5 T-ALL and 5 normal samples and values express the ratio of gene expression of each population / median gene expression. Black boxes highlight BCL-2 family and Caspase genes identified.
